# Oral consumption of Bonito fish‐derived elastin peptide (VGPG Elastin^®^) improves biophysical properties in aging skin: A randomized, double‐blinded, placebo‐controlled study

**DOI:** 10.1111/srt.13634

**Published:** 2024-03-13

**Authors:** Seol Hwa Seong, Young In Lee, Joohee Lee, Jangmi Suk, In Ah Kim, Chaemin Baeg, Jinhak Kim, Ju Hee Lee

**Affiliations:** ^1^ Department of Dermatology Severance Hospital Cutaneous Biology Research Institute Yonsei University College of Medicine Seoul South Korea; ^2^ Scar Laser and Plastic Surgery Center Yonsei Cancer Hospital Yonsei University College of Medicine Seoul South Korea; ^3^ Global Medical Research Center Seoul South Korea; ^4^ R&D Division Daehan Chemtech Co., Ltd. Seoul South Korea

**Keywords:** clinical study, elastin peptide, food derivatives, photoaging, wrinkles

## Abstract

**Background:**

Recent in vitro and in vivo studies have suggested that the elastin peptide improves the skin's biophysical properties, enhancing the proliferation of fibroblasts and elastin synthesis, resulting in anti‐aging properties. Therefore, we conducted a randomized, double‐blinded, placebo‐controlled study to clinically evaluate the effect of elastin peptide intake on human skin.

**Materials and Methods:**

Healthy adult participants (*N* = 100) were randomly assigned to receive a test product containing 100 mg of Bonito elastin peptide (VGPG Elastin^®^) or placebo. In this study, all participants were Asian from Korea. The parameters of skin wrinkles, hydration, and brightening (melanin index) were measured at baseline and 4, 8, and 12 weeks after intervention.

**Results:**

The average skin roughness, maximum peak‐to‐valley values, maximum peak height of the wrinkle, maximum valley depth of the wrinkle, average maximum height of the wrinkle, and eye wrinkle volume improved considerably in the test group compared with the placebo after 12 weeks of intervention. Skin hydration was enhanced, and the melanin index was significantly lower in the test group than in the placebo group. No participant experienced adverse events related to the test product.

**Conclusion:**

Oral consumption of Bonito elastin peptide (VGPG Elastin®) reduced fine wrinkles, enhanced skin moisture, and decreased melanin index without significant adverse effects and may be a promising anti‐wrinkle, anti‐dryness, and anti‐pigmentation treatment.

## 1 INTRODUCTION

Recent years have witnessed a growing enthusiasm for “beauty foods” and their derivatives, owing to their non‐invasive nature, ease of consumption, and fair effectiveness in skin rejuvenation. Collagen peptide, a major component of dermal tissue, has gained popularity for its anti‐aging properties.^1^ Elastic fibers, despite comprising only 3%−5% of the dry weight of adult dermal tissue, are considered the second most crucial dermal component responsible for imparting skin elasticity, resilience, and a healthy dermal environment along with collagen fibers.^2^, ^3^

Skin aging manifests as an intensification of wrinkles, sagging, dryness, and pigmentation, which are particularly evident in photodamaged skin. Ultraviolet (UV) radiation acts as the chief stimulator of reactive oxygen species, pro‐inflammatory signals, and matrix metalloproteinases (MMPs), leading to melanogenesis and degradation and diminished production of the dermal extracellular matrix (ECM), including collagen and elastic fibers.^4^

Photoaging is primarily caused by exposure to UVB and UVA. UVB penetrates the epidermis and superficial dermis, causing direct cellular DNA damage. UVA reaches the deeper dermis and induces oxidative stress, subsequent downstream inflammation, and indirect cellular damage, which involves the mitogen‐activated protein kinase (MAPK) family, AP‐1, and nuclear factor‐κB activation.^5^ Fibroblasts play a key role in maintaining dermal structures by producing collagen and elastic fibers. UV radiation reduces the function of fibroblasts and triggers cellular senescence while upregulating MMPs from keratinocytes and fibroblasts, thereby contributing to skin photoaging.^6^, ^7^ Unlike the epidermis, which undergoes turnover and regeneration, elastic fibers possess a half‐life of >70 years, enduring the deposition of photodamage and degeneration.^8^ Loss of elastic tissue, fragmented elastic fibers and solar elastosis, indicative of elastic fiber degeneration, are critical features of skin photoaging. Consequently, anti‐aging strategies targeting elastic tissue have garnered substantial attention.

Elastin is the core protein that accounts for 90% of elastic fibers and undergoes extracellular aggregation and stabilization through cross‐linking with microfibrils, similar to the synthesis of collagen peptides.^3^, ^9^ Elastin is rich in glycine (30%−45%), alanine, valine, and proline.^3^, ^10^ These amino acid residues are known to interact with elastase and inhibit its functions.^11^ Shigemura et al. have reported that prolyl‐glycine (Pro‐Gly), a major food‐derived elastin peptide, reaches peak serum concentration within 30 min of elastin hydrolysate ingestion and remains detectable even after 4 h in human peripheral blood.^12^ Further, recent in vitro and in vivo data have supported the efficacy of elastin peptides in improving skin health.^13^, ^14^, ^15^, ^16^, ^17^ These peptides enhance elastin synthesis and fibroblast proliferation, reduce epidermal thickness in photodamaged skin, and attenuate UV‐induced cellular damage.^13^, ^14^, ^16^, ^17^

Similar to collagen peptides that have become a popular treatment modality for skin rejuvenation,^18^ elastin peptide shows promise as a therapeutic agent. However, only a few clinical trials have evaluated the efficacy of oral elastin peptide supplementation.^16^, ^19^ Therefore, the present study aimed to demonstrate the efficacy of oral supplementation with Bonito fish‐derived elastin peptide (VGPG Elastin^®^) in improving skin's biophysical properties, particularly focusing on skin wrinkles, dryness, and pigmentation.

## 2 MATERIALS AND METHODS

### 2.1 Test products

The test product contained 100 mg of Bonito fish (*Katsuwonus pelamis*)‐derived elastin peptide (VGPG Elastin^®^, Daehan Chemtech, Seoul, Korea), along with maltodextrin, silicon dioxide, and magnesium stearate. Additionally, the total average molecular weight of the elastin peptide was 582 Da. The placebo contained the same ingredients without elastin peptide. The test product and placebo were administered daily in a capsule form with a small volume, approximately 100–120 mL, of water every morning.

### 2.2 Study design and participants

This clinical trial incorporated a randomized, double‐blinded, placebo‐controlled design. All participants were assigned to either the test or placebo group in a 1:1 ratio, with stratified randomization according to age and sex. The timeframe of this study ranged from December to April, minimizing the influence of UV exposure. A total of four assessments were conducted, with the baseline visit at week 0 and follow‐up visits at weeks 4, 8, and 12. Healthy volunteers with a wrinkle score of ≥3 on the 10‐grade crow's feet photo scale,^20^ and skin hydration <50 arbitrary units measured using a Corneometer (Courage & Khazaka Electronic, Köln, Germany) on both cheeks, were enrolled in the study.

All subjects were required not to change their usual habit such as diet, composition of meals, physical outdoor activity, and instructed to write a diet diary for their intake through a mobile application. Regular consumption of foods rich in elastin or collagen, like dried Bonito fish or can, was not allowed. Detailed inclusion and exclusion criteria were established to ensure the safety and integrity of the results in Table 1. Informed consent was obtained from all the participants.

**TABLE 1:** Inclusion and exclusion criteria for enrollment of participants.

Inclusion criteria
Healthy volunteer aged 35–60 years Diagnosis of wrinkles with a score of ≥3 on the 10‐grade crow's feet photo scale Skin hydration < 50 arbitrary units measured using a Corneometer Cooperative in writing a daily diary and available to use a mobile application during the study Informed of the protocol of the study and signed a written informed‐consent form

Exclusion criteria
Diagnosis of major internal disorders (malignancy, infectious diseases, autoimmune disorders, uncontrolled hypertension, diabetes mellitus, or severe systemic illness) History of major mental disorders (schizophrenia, drug addiction, alcoholism, depression, or mental illness) Diagnosis of major skin disorders that affect the assessment of facial biophysical properties (acne, rosacea, rosacea, contact dermatitis, seborrheic dermatitis, atopic dermatitis, cutaneous lupus erythematosus, or active skin diseases) Prominent erythema or telangiectasia, moles (nevi) at the measuring skin area. History of smoking within a year History of allergies to cosmetics or foods containing elastin peptide or an ingredient in the test product History of dermatologic procedures (Botox, fillers, or laser therapy) within the past 6 months Skincare therapy using Light Emitting Diode devices or peeling within the past 1 month Use of systemic/topical steroids or retinoids within the past 3 months Use of anti‐wrinkle cosmetic products, including retinol, AHA, or moisture‐rich cosmetic products within the 2 weeks Use of oral supplements containing antioxidants, hyaluronic acids, collagen, Evening Primrose Oil, and high concentrations of vitamin A, C, and E within the 2 weeks Current or previous oral intake of contraceptives, female hormones, anti‐obesity medications, or antidepressants Abnormal liver function Abnormal renal function Current or planning to undergo pregnancy or breastfeeding Current participation in another clinical trial, or previous participation in other wrinkle studies within 1 month Conditions judged by the investigator to be inappropriate for participation in the study

### 2.3 Measurements

The anti‐aging properties of elastin peptide consumption were evaluated with respect to the following three categories: wrinkles, moisture, and skin brightening. After gently washing their faces, the participants rested in a stable environmental condition (room temperature, 20−24°C; humidity, 40−60%) for 30 min. All the participants were asked to restrict water intake 1 h before the assessment.

Skin wrinkles were assessed on the periorbital area (crow's feet) via gross examination in conjunction with Mark Vu (PSI Plus, Suwon, Korea) and PRIMOS Lite (LMI Technologies GmbH, Teltow, Germany) assessments, an optical three‐dimensional non‐contact measuring device. The skin surface descriptors in PRIMOS, average skin roughness (Ra), maximum of all peak‐to‐valley values (Rmax), maximum peak height of the wrinkle (Rp), maximum valley depth of the wrinkle (Rv), and average maximum height of the wrinkle (Rz) were measured to represent skin wrinkles and roughness. The *Z* resolution of PRIMOS that we used was 6 μm. In addition, the eye wrinkle volume was assessed to determine three‐dimensional changes in the volume of elevation during the study period.

Skin moisture was measured using the Corneometer CM 825, which detects the hydration level below the stratum corneum using electrical capacitance. Skin brightening was analyzed with the melanin index and erythema index using Mexameter MX 18 (Courage & Khazaka Electronic, Germany). Both the value of the Corneometer and Mexameter were measured on the cheek at the point where the lateral canthus and tip of the nose intersect at a right angle. The average value of three stabilized measurements was used.

### 2.4 Safety assessments

The participants who consumed the test product or placebo at least once were included in the safety analysis set. A comprehensive physical examination was performed at each visit. Blood samples were collected from all the participants at baseline and after completion of the clinical trial to measure the complete blood cell count, liver/renal function, and glucose and cholesterol levels. Urine samples were also analyzed to determine any abnormalities. Details of assessment items were listed in the Supplementary information.

### 2.5 Statistical analysis

All the statistical analyses were performed using SAS software (version 9.4 for Windows; SAS Institute Inc., Cary, NC, USA), and *p*‐values < 0.05 were considered statistically significant. All the data were analyzed using descriptive statistics and compared before and after treatment using the paired *t* or Wilcoxon signed‐rank test. Between‐group comparisons were performed using the two‐sample *t*‐test or Wilcoxon rank‐sum test. McNemar's test was used to assess the results of urinalysis after the results were classified as either normal or abnormal. The incidence rate of adverse effects in each group was compared using the Chi‐squared or Fisher's exact test.

## 3 RESULTS

### 3.1 Participant characteristics

**FIGURE 1:**
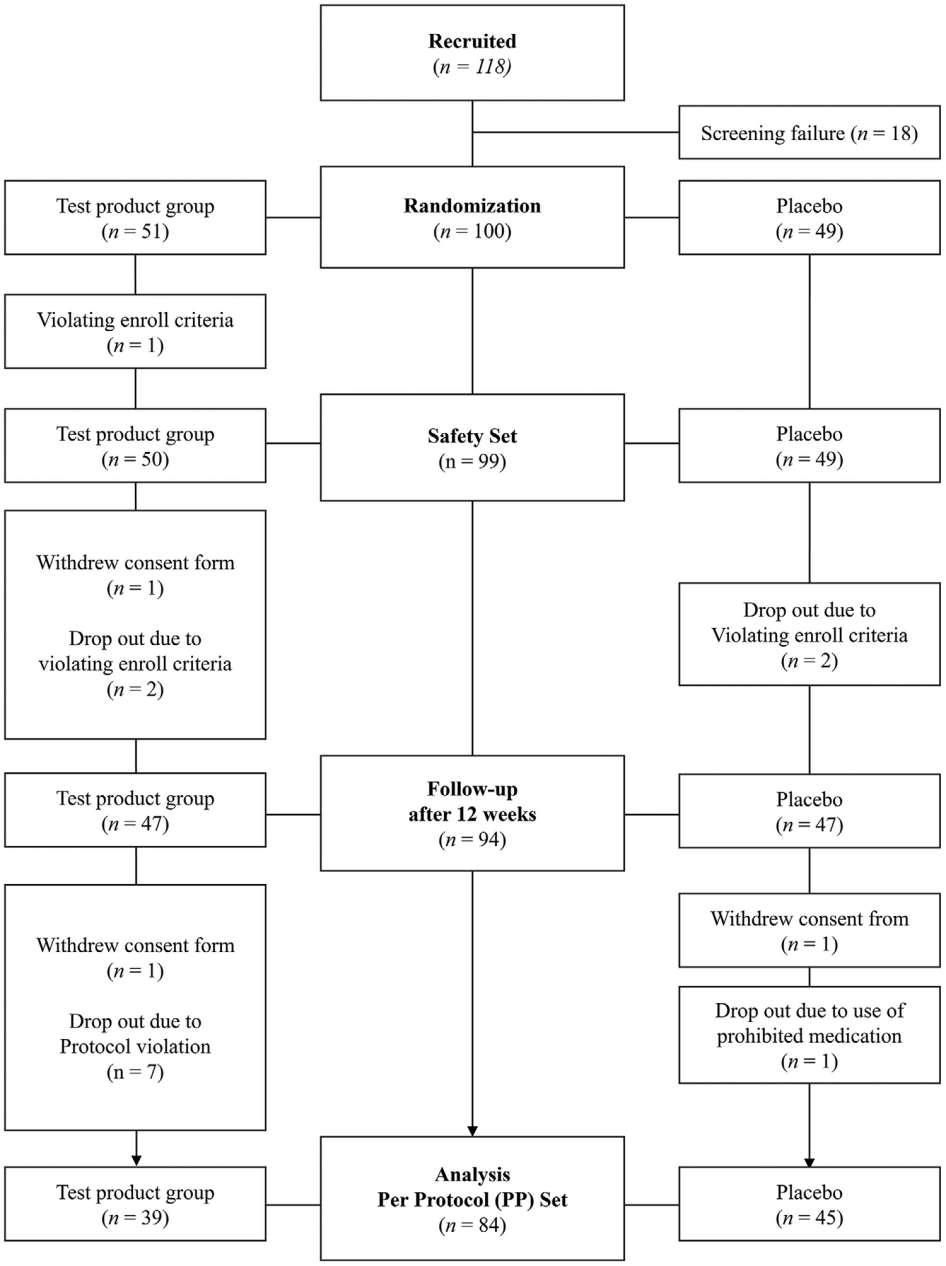
Consolidated standards of reporting trials (CONSORT) flow diagram of the study.

A total of 100 participants were randomized at baseline and allocated to the test (*n* = 51) or placebo (*n* = 49) groups. During the study, 12 participants from the test group and four from the placebo group withdrew consent or were excluded owing to protocol violation. Therefore, the per‐protocol population for efficacy assessment included 84 participants. The safety of intake of the study products was analyzed at least once in 99 participants. Figure 1 depicts the Consolidated Standards of Reporting Trials flow diagram of this study. Baseline demographics and clinical characteristics of the participants are shown in Table 2.

**TABLE 2:** Baseline demographics and clinical characteristics of the per‐protocol study population.

Characteristics	Test group (*n* = 39)	Placebo group (*n* = 45)	*p*‐value
	Mean ± SD or *n* (%)	Mean ± SD or *n* (%)	
Sex			
Male	4 (10.26)	4 (8.89)	1.00^†^
Female	35 (89.74)	41 (91.11)	
Age (years)	46.36 ± 7.12	47.00 ± 6.36	0.60^‡^
Height (cm)	159.81 ± 6.70	160.29 ± 5.98	0.59^‡^
Smoking status			
Never smoker	39 (100.00)	45 (100.00)	N/A
Current smoker	0 (0.0)	0 (0.0)	
Alcohol intake			
Never drinking	21 (53.85)	26 (57.78)	0.86^†^
Quit	1 (2.56)	2 (4.44)	
Drinking	17 (43.59)	17 (37.78)	
Outdoor activity time			
<3 h/week	33 (84.62)	33 (73.33)	0.44^†^
3–5 h/week	5 (12.82)	9 (20.00)	
≥5 h/week	1 (2.56)	3 (6.67)	
Sleeping duration			
<5 h/day	2 (5.13)	4 (8.89)	0.75^†^
5–8 h/day	34 (87.18)	36 (80.00)	
≥8 h/day	3 (7.69)	5 (11.11)	
Use of sunscreen			
0 day/week	12 (30.77)	15 (33.33)	0.55^†^
1−2 days/week	13 (33.33)	10 (22.22)	
3−4 days/week	7 (17.95)	7 (15.56)	
≥5 days/week	7 (17.95)	13 (28.89)	

Abbreviations: N/A, not applicable; SD, standard deviation.

^†^ *p*‐values were determined by Chi‐square or Fisher exact tests.

^‡^ *p*‐values were determined by two‐sample *t* or Wilcoxon rank sum tests.

### 3.2 Effect on skin wrinkles

**FIGURE 2:**
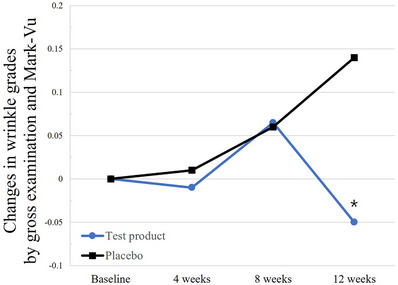
Normalized changes in wrinkle grades by gross examination and Mark‐Vu in the per‐protocol study population. The changes in parameter values from the baseline are shown in arbitrary units. *p < 0.05, **p < 0.001, and ***p < 0.0001 between groups as determined using the two‐sample t or Wilcoxon rank‐sum test.

The periorbital skin wrinkles were assessed via gross examination using a 10‐grade crow's feet photo scale (Grades 0−9) in conjunction with the Mark Vu device, which tended to decrease from 3.92 ± 0.88 at the baseline visit to 3.87 ± 0.92 at week 12 after taking the test product. However, this decrease was not statistically significant (*p* = 0.3047). In contrast, the wrinkle grades of the placebo group increased from 4.19 ± 0.91 at the baseline visit to 4.33 ± 0.92 at week 12 (*p* = 0.0325). The difference between the wrinkle grade changes in the two groups was statistically significant (*p* = 0.0365) (Figure 2).

**FIGURE 3:**
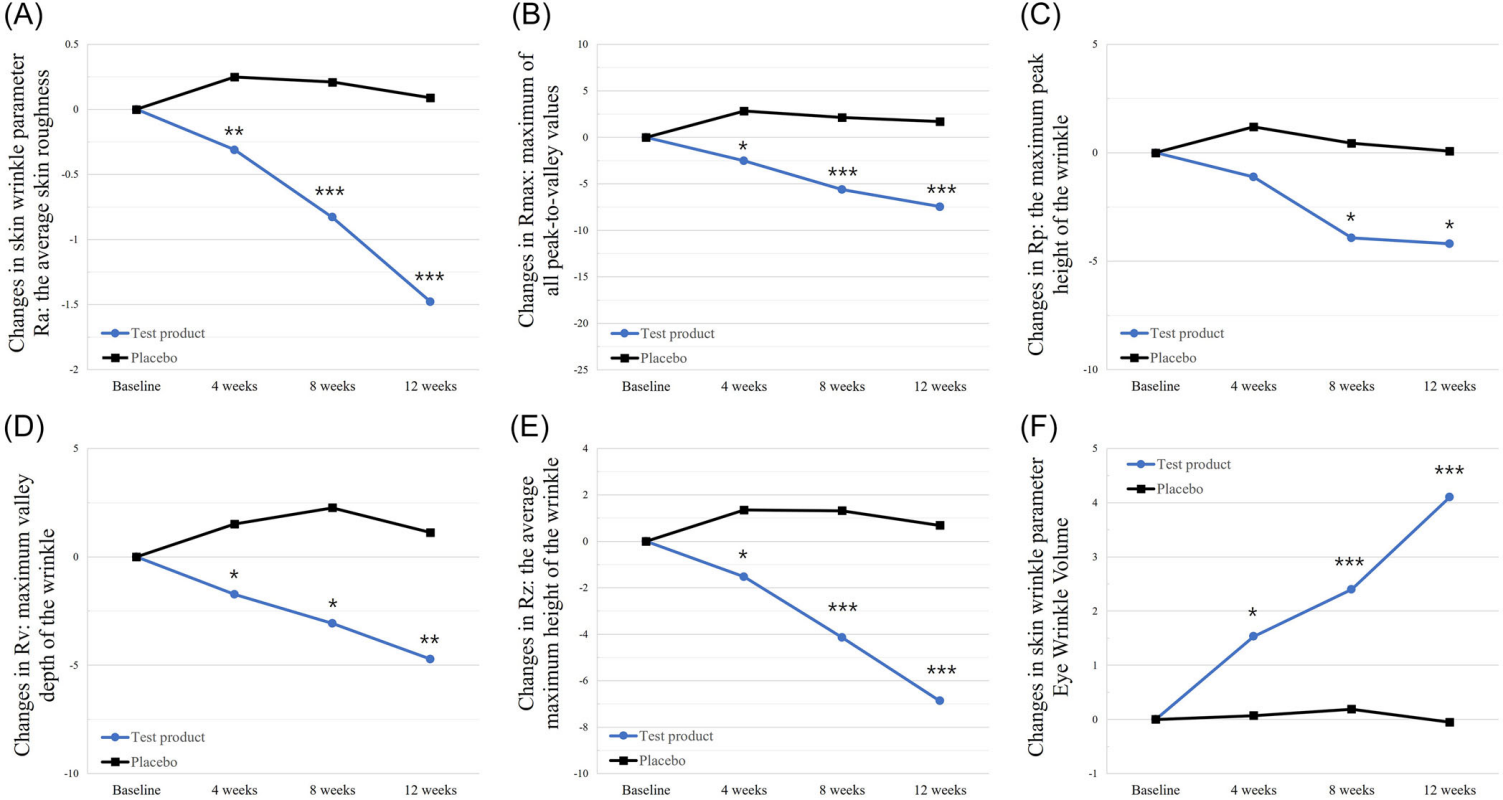
Normalized changes in skin wrinkle parameters of participants in the per‐protocol study population. (A) Average skin roughness (Ra), (B) maximum of all peak‐to‐valley values (Rmax), (C) maximum peak height of the wrinkle (Rp), (D) maximum valley depth of the wrinkle (Rv), and (E) average maximum height of the wrinkle (Rz). Changes in parameter values from the baseline are depicted in micrometers (μm). (F) Eye wrinkle volume is shown in cubic millimeters (mm^3^). *p < 0.05, **p < 0.001, and ***p < 0.0001 between the groups as determined by Wilcoxon rank‐sum test or ANCOVA adjusted baseline and age, drinking, smoking, and sleep.

All the skin wrinkle parameters measured using PRIMOS showed remarkable improvement in the test group at week 12 after the intervention (*p* < 0.001 for Ra, Rmax, Rz; *p *= 0.0061 for Rp; *p* = 0.0004 for Rv), whereas the parameters in the placebo group increased, indicating worsening of wrinkles during the same period. The inter‐group comparison showed evidence of greater anti‐wrinkling properties of the test product over the placebo (*p* < 0.001 for Ra, Rmax, Rz; *p *= 0.0420 for Rp; *p* = 0.0003 for Rv) (Figure 3, Table 3). According to the improved wrinkle parameters, the eye wrinkle volume increased considerably in the test group, that is, from 11.12 ± 3.58 at the baseline visit to 15.22 ± 4.79 at week 12 (*p* < 0.0001), whereas that in the placebo group decreased slightly or remained unchanged. Notably, the volumetric change in eye wrinkles was prominent from week 4, suggesting prompt improvement. The comparison of the changes from the baseline between the two groups was statistically significant during the study (*p = 0.0036* at week 4*; p* < 0.0001 at weeks 8 and 12) (Figure 3, Table 3).

**TABLE 3:** Skin wrinkling parameters of the per‐protocol study population.

		Test group		Placebo group		
Parameter	Time of visit	Mean (SD)	*p*‐value^*^	Mean (SD)	*p*‐value^*^	Intergroup comparison *p*‐value^†^
Ra (μm)	Baseline	19.54 (2.74)		18.81 (3.15)		0.1618
	4 weeks	19.24 (2.71)	0.0008	19.06 (3.14)	0.0508	0.0007
	8 weeks	18.72 (2.63)	< 0.0001	19.02 (2.97)	0.1404	< 0.0001
	12 weeks	18.07 (2.44)	< 0.0001	18.90 (3.04)	0.6090	< 0.0001
Rmax (μm)	Baseline	180.10 (34.13)		180.23 (49.02)		0.4144
	4 weeks	177.59 (32.23)	0.0900	183.08 (48.05)	0.0294	0.0295
	8 weeks	174.50 (33.56)	< 0.0001	182.39 (46.46)	0.0626	< 0.0001
	12 weeks	172.65 (32.90)	< 0.0001	181.93 (48.01)	0.2782	< 0.0001
Rp (μm)	Baseline	104.99 (30.25)		105.69 (49.74)		0.3418
	4 weeks	103.88 (28.93)	0.4099	106.89 (48.92)	0.2246	0.3668
	8 weeks	101.06 (29.79)	0.0022	106.13 (46.82)	0.7381	0.0109
	12 weeks	100.79 (29.75)	0.0061	105.76 (48.88)	0.9510	0.0420
Rv (μm)	Baseline	89.97 (19.36)		88.20 (18.44)		0.6700
	4 weeks	88.25 (18.84)	0.0556	89.72 (18.62)	0.0972	0.0266
	8 weeks	86.90 (18.20)	0.0077	90.47 (16.96)	0.0335	0.0013
	12 weeks	85.25 (17.95)	0.0004	89.33 (17.74)	0.2820	0.0003
Rz (μm)	Baseline	99.44 (13.05)		95.92 (15.51)		0.1462
	4 weeks	97.90 (13.10)	0.0092	97.27 (15.42)	0.0315	0.0015
	8 weeks	95.30 (12.52)	< 0.0001	97.24 (14.56)	0.0484	< 0.0001
	12 weeks	92.57 (12.00)	< 0.0001	96.61 (15.53)	0.4500	< 0.0001
Eye wrinkle Volume (mm^3^)	Baseline	11.12 (3.58)		12.30 (4.10)		0.1319
	4 weeks	12.65 (4.92)	< 0.0001	12.37 (4.15)	0.8463	0.0036
	8 weeks	13.52 (4.23)	< 0.0001	12.49 (4.64)	0.8027	< 0.0001
	12 weeks	15.22 (4.79)	< 0.0001	12.24 (4.23)	0.6131	< 0.0001

Skin wrinkle parameters: Ra, average skin roughness; Rmax, maximum of all peak‐to‐valley values; Rp, the maximum peak height of the wrinkle; Rv, maximum valley depth of the wrinkle; Rz, average maximum height of the wrinkle. SD, standard deviation.

* Intra‐group comparisons: *p*‐values were determined using the paired *t* or Wilcoxon signed‐rank test.

^†^ Inter‐group comparisons: *p*‐values were determined using Wilcoxon rank‐sum test or ANCOVA adjusted base line and age, drinking, smoking, and sleep.

### 3.3 Effect on skin moisture and brightness

**FIGURE 4:**
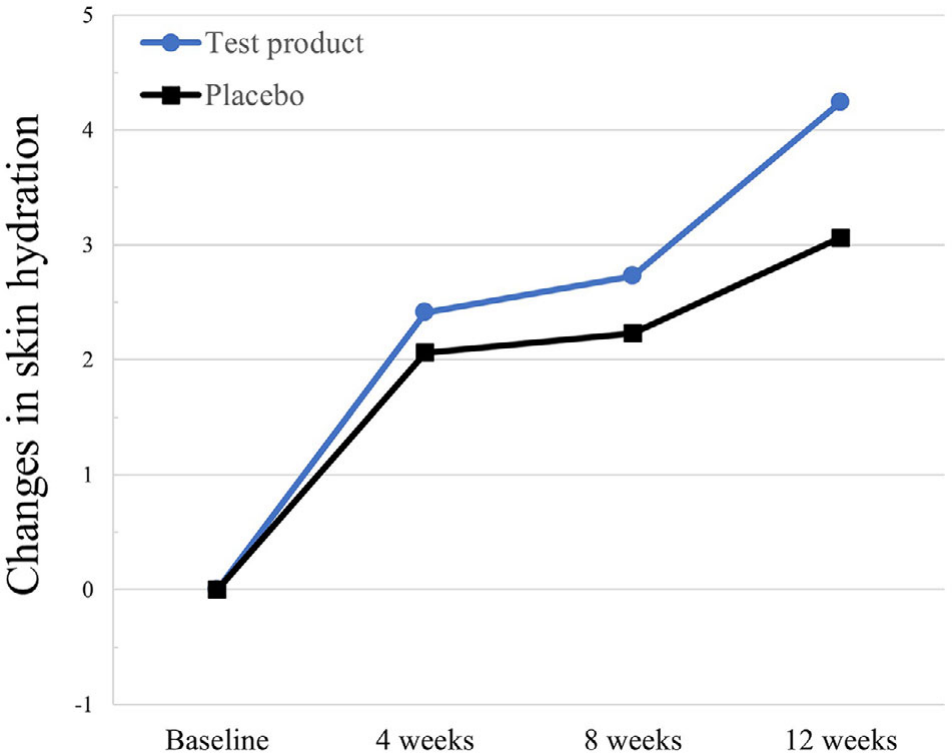
Normalized changes in skin hydration in the per‐protocol study population. Changes in parameter values from baseline are shown in arbitrary units. *p < 0.05, **p < 0.001, and ***p < 0.0001 between groups, as determined using the two‐sample t or Wilcoxon rank‐sum test.

Skin moisture, which was evaluated as skin hydration, improved considerably after the 12‐week intervention compared to its baseline value (from 42.05 ± 4.98 to 46.29 ± 4.55, *p* < 0.0001). Although skin hydration in the placebo group also increased (from 42.78 ± 5.06 to 45.84 ± 4.98, *p* < 0.0001), changes from the baseline were significantly higher in the test group than in the placebo group (*p* = 0.0160) (Figure 4).

**FIGURE 5:**
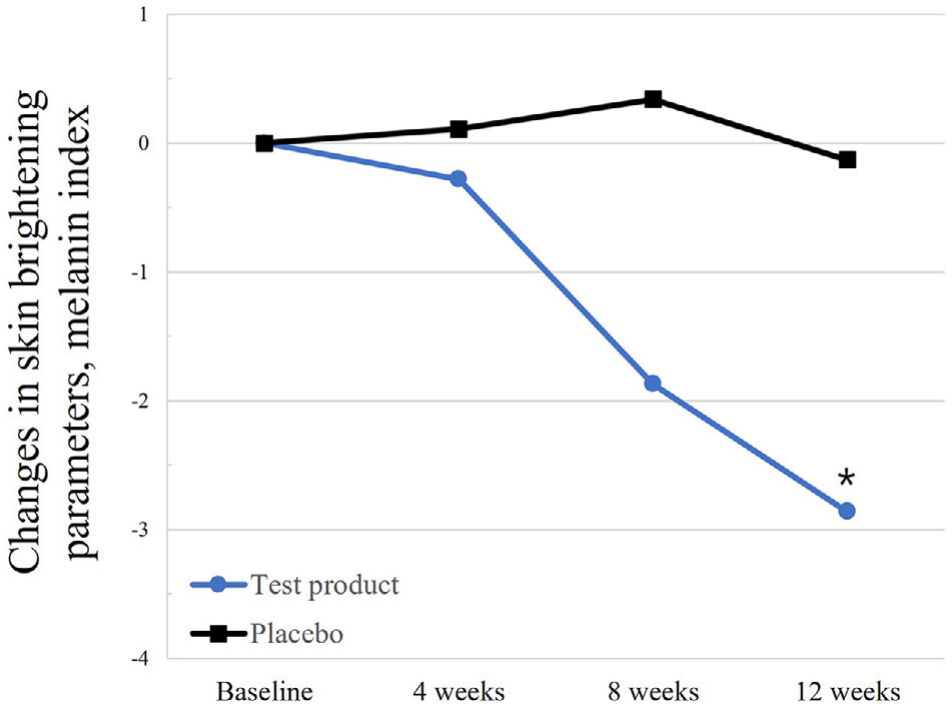
Normalized changes in the skin brightening parameter, that is, the melanin index, in the per‐protocol study population. The changes in the parameter values from baseline are shown in arbitrary units. *p < 0.05, **p < 0.001, and ***p < 0.0001 between groups as determined using Wilcoxon rank‐sum test or ANCOVA adjusted baseline and age, drinking, smoking, sleep.

In the test group, the melanin index presented a greater reduction at week 12 compared with that at the baseline (from 112.74 ± 30.39 to 109.88 ± 28.75, *p* = 0.0181), whereas the index in the placebo group presented no substantial change (from 112.69 ± 29.37 to 112.55 ± 29.50, *p* = 0.9047). A comparison of changes from baseline between the two groups revealed significant differences (*p* = 0.0303) (Figure 5).

Concerning the erythema index, there was no notable difference at baseline between the values of the two groups (295.08 ± 59.59 in the test group and 293.56 ± 61.29 in the placebo group, *p* = 0.7913). Although the erythema index decreased in both groups after 12 weeks, the values showed no statistically significant difference between the two groups (272.75 ± 58.87 in the test group and 277.69 ± 57.59 in the placebo group, *p* = 0.9250).

### 3.4 Adverse effects and laboratory analyses

The test and placebo groups underwent blood and urine analyses, and the results were within the respective normal ranges. Furthermore, statistical analysis of the biochemical data revealed no significant differences between the two groups. No adverse effects were observed in any of the participants throughout the study period. Detailed items of laboratory tests and the reports of adverse events were described in Supplementary information.

## 4 DISCUSSION

In this study, we investigated the effects of Bonito fish‐derived elastin peptide (VGPG Elastin^®^) supplementation in improving the skin's biophysical properties. We found that 12 weeks of oral elastin peptide supplementation improved skin wrinkles and hydration parameters and decreased the melanin index, with significant differences as compared to the placebo group. Elastin is abundant in the arterial bulb of Bonito fish, and VGPG Elastin^®^ is sourced from byproducts generated during can and food manufacturing, ensuring traceable supply chains and contributing positively to the environment.

The elastic fibers in human skin provide resilience and elasticity due to their high extensibility. Characteristically, fine elastic fibers are abundant in the papillary dermis. Microfibrils bridge the basement membrane and dermal plexus of fibers by merging with the elastic fibers, in contrast to the mature elastic fibers that are intermixed with thick collagen bundles in the reticular dermis. Therefore, the role and changes in the nature and placement of elastic fibers can be prominent in the upper dermal layers, where UV radiation can penetrate and damage the skin, as observed with histopathologic examination.

The test group in our study demonstrated improvement in skin roughness and wrinkles (Ra, Rmax, Rz, Rp, Rv) that was objectively measured using PRIMOS, although subjective changes from the baseline were not striking by the gross and Mark Vu examinations. Furthermore, measurement using PRIMOS showed that the eye wrinkle volume, which represents the volume of elevation in the skin surface, was also considerably enhanced, showing skin plumping that contributes to a more youthful look. This smoothness and plumpness of the skin can be explained by increased elastin synthesis and moisturization. As mentioned above, elastic fibers are closely related to the dermo‐epidermal junctions and upper dermal layers. Thus, we can assume that the efficacy of enhanced elastin synthesis by oral consumption of elastin peptide may be foremost at the papillary dermis, which can facilitate detecting the amelioration of fine wrinkles using an objective tool. However, a noticeable decrease in deep wrinkles, as perceived by the naked eye, requires a considerable enhancement in the ECM and major collagen fibers, which may not be the primary finding of elastin peptide intake alone.

Although there was no statistically significant decrease between wrinkle grades by visual inspection at baseline and the end of elastin peptide treatment, a comparison of the wrinkle grade changes between the test and placebo groups showed a significant difference. The favorable interpretation of these results for elastin peptide arises from the study's timeframe, i.e., from December to April, when skin conditions typically worsen with roughness. However, the consumption of elastin peptide appeared to prevent such deterioration during this season.

As mentioned earlier, we determined that the PRIMOS result is more valid. Visual inspection, even with the assistance of the high‐resolution Mark‐Vu device, exhibited disparities among observers and lacked consistency. In our data, visual inspection did not reveal significant differences or distinct trends, highlighting a contrast with PRIMOS data. This aligns with the findings of a recent systematic review on the reliability and validity of skin‐measuring devices, which identifies PRIMOS as an excellent and reliable device for intra and interobserver assessments.^21^

Nevertheless, elastin peptides, which are composed of repeating motifs of valine‐glycine‐valine‐alanine‐proline‐glycine and Pro‐Gly, can reportedly activate elastin synthesis of human dermal fibroblasts.^12^, ^16^, ^22^ Further, elastin peptide intake inhibits the apoptosis of fibroblasts in the skin and increases fibroblast proliferation, thereby reinforcing the collagen and elastin content.^15^, ^16^, ^17^ Zhang et al. reported decreased expression of MMP‐3 and interleukin (IL)−1α and concurrent upregulation of tumor growth factor β (TGF‐β), which are collagen and elastin synthesis‐related factors, after the oral administration of elastin peptide in mice.^15^ Moreover, senescent cells release various pro‐inflammatory cytokines, such as IL‐1α, IL‐1β, IL‐6, tumor necrosis factor‐α, and MMPs, which are involved in the regulation of collagen and elastin synthesis.^7^ Interestingly, Bonito fish‐derived elastin peptide stimulated the expression of TGF‐β, procollagen type I, and collagen type I while downregulating the protein expression of p‐Smad3/Smad (negative factors of the TGF‐ β/Smad pathway), MAPK signaling pathways involving JNK, Fos, Jun, and MMP‐1, 3, and 9 as well as the above‐mentioned pro‐inflammatory cytokines in UVB‐irradiated human fibroblasts and hairless mice.^13^ These findings align with our clinical data on the positive effect of elastin peptide consumption in improving skin wrinkles and roughness.

Further, the improvement in skin hydration, which contributes to plump, smooth, and moisturized skin, can be explained by increased water content and upregulated expression of hyaluronic acid and hydroxyproline following oral elastin peptide intake in mice, which is similar to the effect of collagen.^15^, ^23^ Oral consumption of elastin peptide was found to modify the mRNA expression of long‐chain base 1, dihydroceramide desaturase 1, elastin, the protein expression of hyaluronan synthase 2, and ceramide synthase 4, which affects skin moisturization in UV‐irradiated HaCaT keratinocytes and hairless mice.^13^ Therefore, elastin peptide intake strengthens the skin barrier and enhances hydration, which was consistent with the findings of the present study. However, the changes in skin barrier functions may be subtle, which could not be demonstrated in this study. Thus, specific tools to detect small differences, especially other than the measurement of transepidermal water loss, should be established.^24^

It is compelling that elastin peptide intake exhibits a skin brightening effect. Photoaging skin pigmentation is a sophisticated process that involves senescent cells, including

melanocytes, keratinocytes, and fibroblasts.^7^ Recently, senescent fibroblasts have been implicated importantly in aging‐related skin pigmentation.^25^ Compared to perilesional normal skin, pigmented skin samples obtained from aged individuals and patients with melasma and solar lentigo showed a higher number of senescent dermal fibroblasts, along with a greater release of melanogenic signals such as various growth factors and pro‐inflammatory cytokines from senescent fibroblasts and keratinocytes.^25^, ^26^, ^27^ Subsequently, repeated UV‐triggered inflammation by neighboring senescent cells activates melanocytes, resulting in melanin production and accumulation via a prolonged expression of p53‐mediated proopiomelanocortin, which intensifies α‐melanocyte‐stimulating hormone, melanocortin 1 receptors, and microphthalmia‐associated transcription factor (MITF) signaling pathways.^28^, ^29^ Thus, it is reasonable to infer that therapeutic options aimed at countering skin aging‐induced pigmentation should target senescent fibroblasts, keratinocytes, and melanocytes. In an in vitro study, *skipjack* (Bonito fish) elastin hydrolysate‐derived peptides mitigated UVA‐induced oxidative stress and apoptosis in HaCaT keratinocytes.^14^ Moreover, elastin peptide was found to decrease the melanin content. Treatment of B16F10 cells, a melanoma cell line, using Bonito fish elastin peptide decreased the tyrosinase activity, nitric oxide, and cAMP levels as observed using an enzyme‐linked immunosorbent assay; however, the glutathione levels increased, and the protein expression of protein kinase A, CREB, and MITF reduced. These results were similar to those of an in vivo experiment on hairless mice.^13^ Thus, considering the cross‐talk in photoaging among keratinocytes, melanocytes, and fibroblasts, oral administration of elastin peptide can be a promising option for anti‐wrinkle, anti‐dryness, and anti‐pigmentation treatment. While the erythema index decreased in both groups with no significant differences between them, we attribute this observation to the impact of weather, meaning that skin erythema worsened during the winter due to the dry and cold conditions. Also, prominent erythema or telangiectasia at the measuring skin area was one of the exclusion criteria when the study was conducted.

Not only elastin but also collagen peptides are representative of bioactive peptides that help improve the features of skin aging. However, to our knowledge, a head‐to‐head comparison of the efficacy between collagen and elastin peptides is lacking. Collagen peptides are reported to improve skin moisture, diminish wrinkle formation, and hold melanogenesis, thereby preventing skin photoaging, as demonstrated in vivo and in vitro studies.^30^ Besides, collagen and elastin peptides appeared synergistic for photoaging skin in animal experiments.^15^ Thus, further clinical studies comparing the efficacy of taking collagen, elastin peptides, or both would be warranted.

In this study, a total of 16% of participants dropped out. Specifically, six out of 51 participants in the test group (11%) were excluded from the final analysis, all of which were close to the similarly designed literature. A recent study reported a total dropout rate of 16%, with 14% in the test group.^31^ Another study by Kim DU et al. documented dropout rates of 17% overall, with 21% in the test group.^32^ Additionally, a previous report in our group observed an 18% dropout rate overall, with 11% in the test group.^33^

This study had a few limitations. First, we did not conduct a follow‐up assessment. Therefore, the duration for which the effect of elastin peptide can be sustained after cessation of the test product could not be evaluated. Second, our study utilized only capsules and could not establish an optimal formulation to deliver the elastin. Further research is required to identify the most effective mode of administration. Third, even though all the participants were required not to change their dietary and physical activity habits, we could not strictly restrict the amount of hydration, which may affect the skin's hydration level. Lastly, the study only enrolled a single ethnic group from Korea, and most of the patients were females, which limits the generalizability of the results.

## 5 CONCLUSION

Oral consumption of Bonito fish‐derived elastin peptide (VGPG Elastin^®^) reduced fine wrinkles, enhanced skin moisture, and lowered the melanin index, resulting in skin brightening without significant adverse effects. These findings also suggest that elastin peptide intake may be a safe and excellent measure for other dermatological diseases, such as Riehl's melanosis, melasma, and senile lentigo, as well as aging. Further research remains to elucidate the precise therapeutic mechanisms through which this nutraceutical can impact various dermatological conditions.

## CONFLICTS OF INTEREST STATEMENT

The authors declare no conflict of interest.

## Supporting information

Supporting information

## Data Availability

The data collected during this study are available from the corresponding author upon reasonable request owing to privacy and ethical restrictions.
