# Supplementary material for: Oral consumption of Bonito fish‐derived elastin peptide (VGPG Elastin®) improves biophysical properties in aging skin: A randomized, double‐blinded, placebo‐controlled study
Source: Skin Res Technol. 2024 Mar 13;30(3):e13634. doi: 10.1111/srt.13634 (PMC10938029; doi:10.1111/srt.13634)
Supplement: Supplementary file 1 — Supporting information [file SRT-30-e13634-s001.docx]

**Supplementary information**

**Decision on Analysis Set**

After randomization, one participant was excluded and didn’t take the test product due to violating enroll criteria: “Current participation in another clinical trial, or previous participation in other wrinkle studies within 1 month.”

In the test product group, three didn’t finish the study and follow-up due to withdrawing consent form before week 4 (*n*=1), violating enroll criteria found after week 4 (*n*=2): one with “Current participation in another clinical trial, or previous participation in other wrinkle studies within 1 month”, one who had hypothyroidism and were taking thyroid hormone with “Diagnosis of major internal disorders (malignancy, infectious diseases, autoimmune disorders, uncontrolled hypertension, diabetes mellitus, or severe systemic illness).”

In the placebo group, two participants who had hypothyroidism and were taking thyroid hormone didn’t finish the study and follow-up due to violating enroll criteria found at week 4 (*n*=2): “Diagnosis of major internal disorders (malignancy, infectious diseases, autoimmune disorders, uncontrolled hypertension, diabetes mellitus, or severe systemic illness)”.

After 12 weeks of follow-up, 94 participants completed this study, and 10 participants were not included in the final analysis. The reasons were as follows:

|  | Number | Reason | Remarks |
| --- | --- | --- | --- |
| Test  group | 1 | Withdrew consent form | Withdrawal between week 4 and week 8 |
|  | 2 | Protocol violation | Delayed visit at week 12: 12 days late after visit window* |
|  | 3 | Protocol violation | Delayed visit at week 12: 1 day late after visit window* |
|  | 4 | Protocol violation | Delayed visit at week 12: 12 days late after visit window* |
|  | 5 | Protocol violation | Cosmetic procedure including laser, peeling, etc.: Eyebrow tattoo |
|  | 6 | Protocol violation | Cosmetic procedure including laser, peeling, etc.: Eyebrow tattoo |
|  | 7 | Protocol violation | Use of prohibited medication: taking anxiolytics (alprazolam) |
|  | 8 | Protocol violation | Randomization labeling error |
| Placebo group | 1 | Withdrew consent form | Withdrawal between week 8 and week 12 |
|  | 2 | Protocol violation | Use of prohibited medication: taking steroid (methylprednisolone) |

* The visit window ranged five days before and after the expected date.

**Safety assessment**

The safety set comprised all randomized subjects who received at least one treatment dose and was based on the actual treatment received in clinical trials. The participants who consumed the test product or placebo at least once were included in the safety analysis set. A comprehensive physical examination was performed at each visit.

At each visit, information on adverse events was explored through non-directive questioning. Voluntary reports from participants were allowed at any time during the day through emails or phone calls to the researcher in charge. Whether it felt subtle or insignificant, the participants were encouraged to report all events during or between visits. Findings through medical examinations, blood, and urinary tests during or between visits were also considered. After 8 hours of fasting, the following blood and urinary tests were measured:

Blood tests collected complete blood count (white blood cells, red blood cells, hemoglobin, hematocrit, platelets, neutrophils, lymphocyte, monocyte, eosinophils, basophils), glucose, total protein, albumin, total bilirubin, blood urea nitrogen, aspartate aminotransferase (AST), alanine aminotransferase (ALT), gamma-glutamyl transpeptidase (GTP), creatinine, total cholesterol, high-density lipoprotein (HDL), low-density lipoprotein (LDL), and triglyceride.

Urinary tests collected specific gravity, pH, protein, glucose, ketone, bilirubin, red blood cells, white blood cells, urobilinogen, nitrite. Additionally, urine human chorionic gonadotropin (hCG) was measured in non-menopausal women. The results were within the respective normal ranges. Furthermore, statistical analysis of the biochemical data revealed no significant differences between the two groups.

Compliance for taking test products was checked by surveys and counting remnant capsules for each participant at each visit.

**Reports of adverse events**

We collected the date, duration, severity, outcome, treatment of adverse events, and assessment of causation with the test products. Even for subjects who did not complete the study, for the safety analysis, regular visits with medical evaluation, contacts via emails, and phone calls to collect safety data were encouraged. When the participants reported any discomfort, follow-up of the events was continued until the adverse events disappeared, were treated, or became explainable.

In the test group, three events were reported (chronic periodontitis, anxiety disorder, and chilblains), all of which were irrelevant to the intake of elastin due to the lack of evidence. In the placebo group, three events were reported (shoulder pain, acute vaginitis, and contact dermatitis), all of which were irrelevant to the intake of the placebo capsule. Therefore, we stated that there were no adverse effects related to the oral supplementation of the test product or placebo.
